# Mechanism of Apoptosis Induced by Curcumin in Colorectal Cancer

**DOI:** 10.3390/ijms20102454

**Published:** 2019-05-17

**Authors:** Nor Isnida Ismail, Iekhsan Othman, Faridah Abas, Nordin H. Lajis, Rakesh Naidu

**Affiliations:** 1 Jeffrey Cheah School of Medicine and Health Sciences, Monash University Malaysia, Jalan Lagoon Selatan, 47500 Bandar Sunway Darul Ehsan, Malaysia; nor.ismail1@monash.edu (N.I.I.); iekhsan.othman@monash.edu (I.O.); 2 UniKL MESTECH, A1-1 Jalan TKS1, Taman Kajang Sentral, 43000 Kajang, Malaysia; 3 Laboratory of Natural Products, Faculty of Science, University Putra Malaysia, UPM, 43400 Serdang, Malaysia; faridah@food.upm.edu.my (F.A.); nordinlajis@gmail.com (N.H.L.); 4 Department of Food Science, Faculty of Food Science and Technology, University Putra Malaysia, UPM, 434000 Serdang, Malaysia

**Keywords:** cell death, apoptosis, curcumin, colorectal cancer

## Abstract

Colorectal cancer (CRC) is among the top three cancer with higher incident and mortality rate worldwide. It is estimated that about over than 1.1 million of death and 2.2 million new cases by the year 2030. The current treatment modalities with the usage of chemo drugs such as FOLFOX and FOLFIRI, surgery and radiotherapy, which are usually accompanied with major side effects, are rarely cured along with poor survival rate and at higher recurrence outcome. This trigger the needs of exploring new natural compounds with anti-cancer properties which possess fewer side effects. Curcumin, a common spice used in ancient medicine was found to induce apoptosis by targeting various molecules and signaling pathways involved in CRC. Disruption of the homeostatic balance between cell proliferation and apoptosis could be one of the promoting factors in colorectal cancer progression. In this review, we describe the current knowledge of apoptosis regulation by curcumin in CRC with regard to molecular targets and associated signaling pathways.

## 1. Introduction

Colorectal cancer (CRC) has been identified as the second leading cause of cancer death (832,000 deaths) worldwide with incidence estimation of 16.5 million [1] in 2015. It is among the top three cancers in men following lung and prostate cancer with incidence and mortality rate of 920,000 cases and 456,000 deaths. CRC ranked second after breast cancer in women with 733,000 cases and 376,000 deaths [1]. CRC prevalence is expected to increase by 60%, which attribute over than 1.1 million of death and 2.2 million new cases by the year 2030 [2]. CRC incidence was more prevalent in Europe with 447,136 CRC cases and 214,866 deaths in 2012 [3] followed by USA with 135,430 diagnosed CRC cases in 2017 [4]. However, the incidence of CRC has been reported to be stable in Northern and Western Europe and also USA, but increased drastically in Australia, New Zealand, and Japan [5]. While CRC is uncommon in Africa, Asia, and India [6], the incidence has rapidly increased in the Asia-Pacific region such as China, Thailand, Philippines, Republic of Korea, and Singapore [5].

Despite the use of advanced surgical removal and chemotherapy treatment for CRC, the survival and recurrence rate of CRC patient has not improved although treatment involved multiple approaches [7]. Common drugs used in CRC treatment are anti-vascular endothelial growth factor, VEGF (Bevacizumab), anti-epidermal growth factor receptor, EGFR (Cetuximab or Panitumumab), topoisomerase inhibitor (Irinotecan), and fluoropyrimidines (capecitabine or 5-fluorouracil or also known as 5-FU) [8]. These drugs may either be used separately or in combination. Generally, the known drug combinations in CRC treatment are CAPOX (capecitabine + oxaliplatin), FOLFIRI (Leucovorin + 5-FU + Irinotecan), and FOLFOX (Leucovorin + 5-FU + Oxaliplatin) [9]. Gastrointestinal ulcerations, kidney damage [10], hearing loss and balance [7] [11,12,13], tumor resistance [14,15], hypertension, fatigue, hand-foot skin reaction, diarrhea [16], and nausea are the list of adverse side effects associated with the chemotherapy treatment. This highlight the need for safer and effective approaches focusing on the discovery of new compounds among natural sources with chemotherapeutic properties. Several studies reported that bioactive compounds isolated from plants demonstrated anti-proliferative and anti-carcinogenic effects towards colon cancer cells [17].

Curcumin, a bioactive compound which is found naturally as turmeric derivative is one of the most frequently used and widely researched phytochemical to have anti-cancer and chemopreventive activity [18]. It was first isolated by Vogel in 1815 [19] with a molecular weight of 368.37 g/mol and C_21_H_20_O_6_ as its molecular formula [18]. Curcumin is an oil-soluble coloring compound, readily soluble in acetic acid, ketone, alkali and chloroform. In contrast, curcumin is insoluble in water at acidic or neutral pH [20]. Curcumin also consists of fats, minerals, carbohydrates, proteins, and has a moisture content [18]. Curcumin may pass through the plasma membrane easily and spread throughout the membranes of ER, mitochondria and nucleus once it is inside the cell due to its hydrophobic characteristic [21]. Its effect on cell membranes during apoptotic cell death was immediate and the loss of membrane integrity is partly reversible enable cells to recover at a faster rate [22]. The CH_2_ group or OH group of the β-di ketone and phenolic OH groups play an important role in biological activity of curcumin [23]. Its beneficial properties include anti-inflammatory, antioxidant, chemo-therapeutic, anti-mutagenic, chemo-preventive, anti-metastatic, and anti-angiogenic [24]. Its targets multiple mechanism of cell death such as transcription factors, membrane receptors, kinases, cytokines, and pathways [25]. At the molecular level, curcumin inhibits colorectal cancer stem cell growth through regulation of self-renewal associated signaling pathway, regulation of growth factor, epigenetic modification, cell cycle arrest, apoptosis, and regulation of structural integrity [26].

## 2. Apoptosis in Normal Colonic Epithelia

Apoptosis refers to a highly regulated physiological process of cell death, responsible for removal of cells that are no longer needed, highly damaged, mutated, and/or aging and unrepairable, thus preserving cells integrity and organism as a whole. The physiological process is described by biochemical and morphological changes such as shrinkage of nuclei, nuclear fragmentation and chromatin condensation, dilated endoplasmic reticulum, cell and cytoplasmic shrinkage, dynamic membrane blebbing, and loss of adhesion to the neighboring cells or to the extracellular matrix components [27,28]. Imbalance of apoptosis either excessive or less may lead to pathogenesis of a wide array of diseases such as autoimmunity, ischemia, neurodegeneration and cancer [29]. Stimulation of apoptosis occurred upon exposure to harmful carcinogens or mutagenic agents, viral infections and ultraviolet radiations. Commitment for cells to undergo apoptosis is triggered by extracellular or intracellular signals, which includes activation of caspase family involving two different pathways. It is either the intrinsic pathway that mainly influences mitochondria permeability, which is also known as the mitochondria pathway, or the extrinsic pathway where involvement of direct interaction between the death ligand and its death receptor, or also referred to as the death-receptor mediated pathway [30,31].

Apoptosis plays a vital role in maintaining normal colonic epithelia. The normal structure of colonic crypts is preserved by a dynamic equilibrium between apoptosis at the top of the crypt and cell proliferation at the base [32,33,34]. Differentiated colonic cells that divide rapidly migrates to the top of the colonic crypt are discard into the colonic lumen via apoptosis, while the slow dividing colonic cells remain at the bottom [35]. Either spontaneous apoptosis or induced apoptosis may occur in colonic epithelia depending on the circumstances [32]. Spontaneous apoptosis occurs continuously in unstressed and normal colon, while stress-induced apoptosis occurs in response to DNA damage either by drugs, radiation or viral infection [36]. There is an increased evidence displaying the disturbance between the balance of cell growth and apoptosis rates during CRC formation. Failure and inhibition of apoptosis may cause an imbalance of intestinal epithelial cell homeostasis, which links to the formation of CRC and its poor response to radiation and chemotherapy [37]. The mutation in the tumor suppressor genes (*APC*, *TP53,* and *SMAD4/DC4*) and oncogenes (*KRAS*, *PIK3CA,* and *BRAF*) will result in an inefficient of apoptosis mechanism. Failure in eliminating mutated colonic cells, while a continuously increasing proliferation rate is an early sign towards developing CRC carcinogenesis. The accumulation of continuous mutated colonocytes will lead to the formation of early adenoma that later, may progress into adenocarcinoma and eventually late stage of CRC [38].

## 3. Dysregulation of Apoptosis in Colorectal Cancer (CRC)

The continuous genomic modification due to genomic instability in CRC may cause alterations in the genes which have been observed to be involved in regulating apoptosis [39]. Greater inhibition of apoptosis has been found to be associated with the transition of colorectal epithelium to CRC [40]. The decrease in apoptosis was more prone in patients with carcinoma (n-34) sporadic adenomas (n-26) and familial adenomatous polyposis (n-25) compared to patients with polyps [41]. *Adenomatous polyposis coli* (*APC*) is one of the genes that have been well characterized and is linked to the apoptosis disruption of the intestinal epithelium and CRC progression [42].

Loss of function of the *APC* gene due to mutation was among the earliest events in the early development of sporadic CRC progression pathway [43,44,45]. About 80% of sporadic colorectal adenomas are involved with *APC* mutation [46]. APC plays a vital role in controlling colon cancer cell growth via regulation of gene transcription mediated by β-catenin [47]. Wild type APC induces degradation of β-catenin, a protein that forms a complex with cadherin via ubiquitin-mediated proteasomal degradation. The truncated APC proteins prevent the targeting of β-catenin for degradation, promote stabilization of nuclear β-catenin [48], and causes continuous activation of the Wnt pathway [30] which lead to disruption in the apoptotic machinery and progression of CRC. This β-catenin migrates to the nucleus and binds to the transcription cofactor T-cell factor/lymphoid enhancement factor (TCF/LEF) in which it affects the expression of over 500 genes, including genes responsible for apoptosis, cell migration, stem cell differentiation, cell proliferation, and cellular growth [49,50,51] and may also serves as an intracellular signal transducer in the Wnt signaling pathway [52,53]. The activation of the β-catenin may also reduce the expression of apoptosis initiator pro-caspase 9, effector caspase 3 and 7, and cytochrome C expression [54]. Disturbance in the equilibrium between pro- and anti-apoptosis proteins may prevent the colonic cells from undergoing apoptosis [55] and may gain resistant to apoptotic stimuli such as radiotherapy and chemotherapeutic drugs [55,56]. Accumulation and increase of β-catenin may also prevent the colonocytes from migrating out of epithelial crypts to be shed off [38] and remain at the colonic crypt.

## 4. Curcumin Overview

Curcumin, a yellow pigment bioactive compound isolated from turmeric or also known as *Curcuma longa*, was a common spice used in Indian cooking and ancient medicine, Ayurveda [46]. This yellow pigment has widely been used for centuries in the treatment for intermittent fevers, diarrhea, constipation, skin diseases, leukoderma, intestinal worms, inflammation, urinary discharges, dyspepsia, amenorrhea, arthritis, rheumatism, body ache, colitis, biliousness, hepatic disorder, and hepatitis [57,58]. Curcumin, also known as diferuloylmethane [1,7-bis-(4-hydroxy-3- methoxyphenyl)-1,6-heptadiene-3,5-dione], has been extensively studied on its anti-cancer properties. Curcumin has been found to inhibit carcinogenesis in preclinical trial performed on various cell lines, including prostate, pancreatic, ovarian, oral epithelial leukemia, hepatic, breast, cervical, gastric, and colon cancer [59,60,61]. Its anti-cancer properties were not limited to cell lines, but have been documented on human and animals models [62]. Curcumin has the ability to modulate several cellular signaling pathways associated with carcinogenesis and cancer growth inhibitor such as suppression of angiogenesis and induction of apoptosis in several cancers, including hepatic [63], human mantle cell lymphoma, brain, breast, ovarian, bone, leukemia, and bladder [59,60]. Curcumin may potentiate apoptosis in CRC due to its ability in inducing reactive oxygen species (ROS) production [64], downregulation of inflammatory pathway mediated by nuclear factor kappa-light-chain-enhancer of activated B cells (NF-κB) [65] and cyclooxygenase-2 (COX-2) [66], inhibits activating protein-1 (AP-1) [65], activation of c-Jun N-terminal kinases (JNK) [67], activation of caspase 3 [68], and the release of cytochrome C. Curcumin has been reported to induce apoptosis in HCT-116 colon cancer cells via increased activation of Bax, caspase 8, caspase 3, caspase 9, and poly(ADP-ribose) polymerase (PARP) [69].

In this review, we describe the apoptosis regulation of curcumin emphasizing on the molecular targets and the underlying pathways involved in CRC. It has been suggested that apoptosis induction by curcumin on colon cancer to be associated with the extrinsic and intrinsic pathway, ROS involvement and endoplasmic reticulum (ER) stress [70,71].

## 5. Molecules Target and Signaling Pathway of Apoptosis Induced by Curcumin

**Figure 1:**
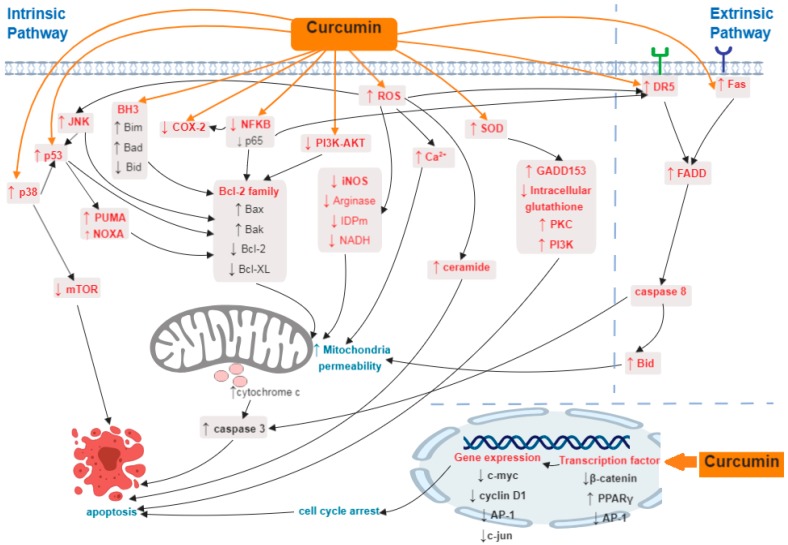
Summary of induction of apoptosis by curcumin in colorectal cancer (CRC). Curcumin induces apoptosis in CRC through multiple target molecules and associated signaling pathways. Curcumin inhibits nuclear factor kappa-light-chain-enhancer of activated B cells (NF-κB)and cyclooxygenase-2 (COX-2), down-regulates transcription factor β-catenin and activating protein-1 (AP-1), suppresses anti-apoptotic proteins and increase reactive oxygen species (ROS), superoxide dismutase (SOD), and pro-apoptotic proteins and also up-regulates Fas and death receptor 5 (DR5) receptor. Molecules in red represents the main targets of apoptosis while molecules in black are the downstream targets of the molecules labelled in red.

One of the anti-cancer effects of curcumin on CRC cells is widely known to be associated with the activation of the apoptosis pathway. Extensive studies on the underlying mechanism of apoptosis by curcumin in CRC involved multiple molecular targets including enzymes (such as COX-2, superoxide dismutase (SOD)), transcription factors (such as β-catenin, NF-κB, AP-1, peroxisome proliferator-activated receptor gamma (PPAR-γ), and p53), ROS, Bcl-2 family members (such as Bak, Bcl-2, Bax, and Bcl-xL), BH3 proteins (such as Bim, Bad, and Bid), protease enzymes (such as caspase 3, caspase 8), death receptors (such as death receptor 5 (DR5), Fas), and other important signaling pathways such as p53, phosphatidylinositol 3-kinase/protein kinase B (PI3K/AKT), JNK, and ER stress (Figure 1).

### 5.1. The Tumor Necrosis Factor (TNF) Ligand Family (TRAIL), Death Receptor 5 (DR5) and Caspase 8

Changes in the apoptosis-regulating cytokines are one of the factors contributing to the disruption and resistance towards apoptosis [72,73]. The tumor necrosis factor (TNF) ligand family (TRAIL) consists of cytokines and it serves as apoptosis mediators [74]. Binding of specific pro-apoptotic membrane receptors of TNF receptor family (receptor Fas/CD95, receptor DR4 and DR5) [75], through ligands such as (FasL23/CD95L), and Apo2 ligand TNF-linked apoptosis-initiating binding groups (Apo2L/TRAIL) [75], activates the intracellular apoptotic machinery via extrinsic pathway. The binding of the “death” ligand to its receptors leads to the formation of death-inducing signaling complex (DISC), followed by the activation of caspase 8, which activates caspase 3 and consequently initiates apoptosis [42]. Caspase 8 might also be involved with the intrinsic pathway by cleaving Bid (a pro-apoptotic member of Bcl-2 protein), causing continuous release of cytochrome C [76,77].

Extrinsic apoptosis regulation in colon carcinoma cell lines has been found to be involved with the immune system regulation [74,78] and it might be associated with TRAIL and Fas signaling pathway. Overexpression of FasL [79,80,81,82], accompanied by downregulation of FasR expression [72,73,83,84] as well as abnormality in the Fas-mediated apoptosis signaling pathway [84], may lead to inactivation of the “death ligand” apoptotic pathway [74,78,85]. Colorectal cancer cells with inactive ligands may acquire a state of immunity where the cells are able to avoid the cytotoxic immune system signal, capable of invading the immune system, thus gaining survival advantage and metastatic potential [72,73,74,78,79,80,81,82,83,84]. This event might be explained by the “Fas-counterattack hypothesis” [79,84,86,87]. Expression of FasL was found during the early stage of adenoma to carcinoma sequence of CRC [88]. In addition, most colon cancer cells lines with positive FasR happen to be resistant to Fas-mediated apoptosis as an indicator of abnormality in the Fas-mediated signaling pathway [84].

Other than disruptions in Fas-mediated apoptosis, defect in TRAIL-mediated apoptosis pathway may also contribute to the colorectal cancer progression. Abnormality in the transport of DR4 receptors, redistribution of DR in lipid raft, caspase 8 mutation and inactivation of caspase 10 and associated protein are the lists of contributing factors for the TRAIL-mediated resistance towards apoptosis [89,90,91]. Downregulation of death receptor-DR4 and DR5 (also known as TRAILR1 and TRAILR2, respectively) expression; and up-regulation of the decoy receptors (DcR)—DcR1 and DcR2 (also known as TRAILR3 and TRAILR4) accompanied by increased expression of TRAIL will disrupt the apoptotic signaling pathway [92]. Binding of TRAIL to the decoy receptor instead of DR may prevent or suppress colon cancer cells from undergoing apoptosis via the extrinsic pathway [92].

This scenario is prone in more aggressive tumor type and worse clinical outcome [87]. There is also other evidence that the TRAIL regulation might be involved in transforming growth factor β (TGF-β)-induced cell death [93]. Mutational inactivation of the TGF-β receptor (TGFβR1) occurs in 30% of CRC causing disruption in TGF-β signaling [41].

The other mechanism involved in the regulation of the extrinsic pathway is the cellular flice-like inhibitory protein (c-FLIP) [6]. c-FLIP is similar to Fas-associated protein with death domain (FADD), a DD-containing protein which may competitively bind to FADD during the DISC formation process instead of the DD domain of the DRs [92]. This c-flip protein isoform shows an almost identical structure to pro-caspase 8 and may serve as potent inhibitor of the extrinsic apoptotic pathway [92]. Curcumin may have the ability to regulate apoptosis extrinsic pathway in CRC by Fas-mediated apoptotic pathway, activation of caspase 8 and binding of TRAIL to its DR. Curcumin was reported to up-regulate the DR5 protein, the receptor required for the TRAIL-induced apoptosis in HCT-116 and HT-29 colon cancer cells [94]. Up-regulation of TRAIL-induces apoptosis in cancer cells was by reactive oxygen species (ROS)-mediated DR5 activation [94], and by suppression of NF-κB through inhibition of IκBα phosphorylation [95]. In addition, curcumin was found to enhance caspase 8 activation which initiates Fas-mediated apoptotic pathway [71,96]. The role of caspase 8 in triggering extrinsic apoptotic pathway is well characterized. A complex is formed between pro-caspase 8 with Fas ligand which is connected via FADD forming DISC, and the activation of caspase 8 by reciprocal cleavage initiates executioner caspase 3, caspase 7, or Bid. Activation of caspase 3, caspase 7, and cleavage of Bid were also observed in HT-29 cells treated with curcumin [71]. The translocation of cleaved Bid to the mitochondria facilitate the release of cytochrome C and subsequently induce apoptosis [97]. The same Fas-mediated apoptotic pathway in colon cancer cells was also observed in human melanoma cells where curcumin induced apoptosis was mediated by a FasR/caspase 8 pathway [98].

### 5.2. Bcl-2 Family Member

One of the keys to apoptosis regulation is the anti-apoptotic B-cell lymphoma-2 (Bcl-2) which controls the release of pro-apoptotic factors that affect mitochondria outer membrane permeability [99]. Various cancer types and malignancies including CRC have been linked to the abnormal expression of Bcl-2 [100]. Dysregulation of colonic epithelial cell apoptosis by abnormal expression of Bcl-2 might leads to colorectal carcinogenesis. It is estimated about 30%–94% of human CRC displayed overexpression of Bcl-2 [92]. Higher expression of Bcl-2 was observed in colorectal adenomas than carcinoma suggesting that Bcl-2 might be linked to the early stage of CRC development [42,101,102,103,104,105,106,107,108]. Most colonic adenomas displayed a high level of Bcl-2 protein throughout the neoplastic epithelium [103,105,106,107,108,109] while non-neoplastic polyps showed a normal pattern of Bcl-2 expression [103,110,111]. It was suggested that overexpression of the Bcl-2 correlated with the transition between hyperplastic epithelium to adenomas [42]. The Bcl-2 protein is normally expressed along the crypts of normal colonic epithelium proportionate to the stem cell compartment where the apoptosis rate is low [112]. The highest Bcl-2 expression is observed at the base and the lowest at the tip of the crypts [113]. Continuous event of p53 mutation along the progression of CRC carcinogenesis is one of the factors affecting the Bcl-2 expression. This scenario explained the decrease in apoptosis at the late stage of colorectal cancer [108,114] and highlights the role of Bcl-2 in the early stage of CRC [104]. In addition, elevated expression of Bcl-2 and deficiency in Bax might cause apoptosis-resistance in colon adenocarcinomas [70]. Curcumin was found to increase Bax expression and decrease Bcl-2 in colon adenocarcinoma through the phosphorylation at Ser15 and activation of p53 [115]. It is suggested that activation of p53 by Ser15 phosphorylation transactivates Bax expression. Increased in the Bax expression may affect Bcl-2/Bax or Bcl-xL ratio thus favoring colon cancer cell towards apoptosis. While the mechanism of curcumin modulates the ratio of anti-apoptotic and pro-apoptotic protein in inducing apoptosis of colon cancer cells remain unclear, similar findings has been reported in breast cancer cells [116]. Suppression of Bcl-2 level and up-regulation of Bax by curcumin has also been observed in other colon cancer cells such as HCT-116 [96] and COLO-205 cells [117]. Bcl-2 suppression may influence the efflux of Ca^2+^ through the ER membrane thus inducing apoptosis [118,119]. Down-regulation of Bcl-2 resulted in the increase production of Ca^2+^ in the ER [120,121,122]. Massive movement of Ca^2+^ from the ER to mitochondria leads to the opening of the mitochondrial permeability transition pore (mPTP) and mitochondrial outer membrane permeabilization (MOMP) [123,124]. In contrast, increased in Bcl-2 expression has been reported to interfere with the generation of oxygen radicals in mitochondria thus preventing the opening of mPTP and MOMP [119]. Curcumin was found to induce apoptosis in RC cells by rapid and continuous increase in Ca^2+^ via the down-regulation of Bcl-2 protein [117]. The disruption of mitochondrial outer membrane lead to the release of cytochrome C and subsequently undergone apoptosis [99,125,126,127,128].

### 5.3. Nuclear Factor-kappa B (NF-κB)

Curcumin as an anticancer agent has been reported to induce apoptosis, reduce survival and able to down-regulate Bcl-2, VEGF, cyclin D1, pro-oncogenic factors and NF-κB in colon cancer cells [129,130]. NF-κB has been widely studied and its implication in CRC involved in the regulation of metastasis, angiogenesis, inflammation, cell proliferation and apoptosis [131]. The NF-κB family member consists of c-Rel, RelB, Re1A (p65), NF-κB1 (p50/p105), and NF-κB2 (p52/p100) with each of them sharing the common Rel homology domain (RHD:300 amino acid). This domain facilitates the binding between DNA and its IκBs which comprise of IκBα, IκBβ, IκBε, IκBγ, IκBζ, p100, and p105. NF-κB in an inactive state remains attached to the IκB, which functions as intracellular NF-κB inhibitor [132,133] and may be activated by ROS, growth factors, viruses, mitogens, pro-inflammatory cytokines, environmental stress, bacterial product and chemotherapeutic drugs [134,135,136]. Once activated by phosphorylation and degradation of the IκB, the NF-κB migrates to the nucleus and attached to the κB site in the promoter or enhancer regions of the “critical genes” that regulate innate and adaptive immune responses, invasion, metastasis, angiogenesis, cell proliferation, cell survival, and apoptosis [134,137,138,139,140,141]. Curcumin, a naturally yellow occurring phenolic compound, has shown to inhibit NF-κB-luciferase activity in HT-29 colon cells and able to suppress the lipopolysaccharide (LPS)-induced phosphorylation of IκBα [142,143]. Continuous activation of NF-κB in CRC has been reported [144,145,146,147], without activating mutation of NF-κB [141]. Poor survival outcomes [148], chemoresistance [129,130,145,149,150] and metastasis [146,151] in CRC are associated with the continuous activation of NF-κB. Suppression of apoptosis and continuous inhibition of JNK activation in CRC occurs as the results of the constitutive activation of NF-κB that targets the anti-apoptotic genes via the p65/Re1A domain to X-linked inhibitor of apoptosis (XIAP), A20 and Bcl-xL [70]. Collect and Campbell [152] reported that curcumin treatment induced apoptosis in HCT-116 cells via activation of JNK and inhibition of NF-κB. Suppression of NF-κB is through inhibition of p65 expression, NF-κB-dependent transcriptional activity and expression of NF-κB-dependent anti-apoptotic genes. In contrast, overexpression of p65 potentiates curcumin-induced apoptosis mediated by JNK activation, although it is noted that inhibition of p65 leads to the sustained activation of JNK. The activation of JNK is independent of NF-κB transcriptional activity suppression and is not associated with the repression of NF-κB anti-apoptotic target genes [152]. In addition, curcumin was found to suppress growth and induce apoptosis of colon cancer cells via inhibition of hepatocyte growth factor receptor (c-MET), specificity protein (Sp) transcription factor such as Sp1, Sp3, Sp4, and Sp-regulated genes including *Survivin*, cyclin D1, Bcl-2, and NF-κB (p65 and p50) [153].

### 5.4. Wnt/β-catenin

β-catenin transcription factor plays a critical role in the carcinogenesis of CRC due to the mutation in the *APC* gene [154]. Loss of function in the APC protein observed in most of colorectal carcinomas may affect the β-catenin degradation [155,156,157] and pool [158]. The APC regulates β-catenin degradation through the regulation of β-catenin phosphorylation, localization and ubiquitination [159]. The APC regulates the scaffold of Axin complex thus regulating the β-catenin phosphorylation. Upon phosphorylation, APC releases the phosphorylated β-catenin from Axin complex for ubiquitination and degradation [159,160]. The truncated APC proteins however, may prevent β-catenin degradation due to inability in releasing β-catenin from the Axin complex or lacking the Axin binding domains [159,160]. This result in significant increase of β-catenin pool that might be involved in Wingless/Wnt signaling pathway, associated with the cell membrane, existing in the cytoplasm or associated with gene regulation [158]. All of this pool might be directly or indirectly contributed to the apoptosis disruption in CRC. Truncation in the APC proteins affect the β-catenin degradation thus activating the Wnt signaling pathway that regulates expression of genes associated with apoptosis and cell cycle such as *c-myc*, cyclin-D, *AP-1* transcription factor, *c-JUN* and *fr-1* [161,162]. Curcumin inhibits Wnt/β catenin pathway by suppressing c-myc expression, induce caspase 3 mediated cleavage of β-catenin, E-cadherin, and APC, which were linked to apoptosis and G2/M phase arrest in HCT-116 colon cancer cells [163,164]. Other studies on colon cancer reported curcumin inhibits β-catenin/Tcf signaling in SW480 and HCT-116 due to the decreased levels of nuclear β-catenin and Tc-4 protein [165]. Besides, curcumin given orally to colon cancer mice carrying *APC* gene mutation (Min/^+^) increased the enterocyte apoptosis and decreased expression of β-catenin oncoproteins [166]. Moreover, curcuminoids treatment on HCT-116 colon cancer cells inhibits JMJD2C, a histone demethylase which forms a complex with β-catenin that are commonly overexpressed in CRC [167]. In addition, tetrahydrocurcumin (THC) a metabolite of curcumin shown to reduce Wnt-1, β-catenin, and phosphorylation of serine/threonine kinase glycogen synthase kinase-3 (GSK-3) in colonic tissue of Azoxymethane (AOM)-induced colon carcinogenesis [168].

### 5.5. Peroxisome Proliferator-Activated Receptor-γ (PPARγ)

Peroxisome proliferator-activated receptor γ (PPARγ) is a ligand binding transcription belong to the nuclear receptor family [169] which function includes regulation of lipid cell growth, metabolism, immune function, differentiation and apoptosis [170]. It plays a role in gene transcription regulation by binding to the promoter region of DNA sequence target genes or also known as peroxisome proliferator response elements (PPREs) [171]. Activation of PPARγ has been understood to induce growth arrest and differentiation markers of human colon cancer cells [172,173,174]. PPARγ may also serve as a tumor suppressor, as suggested by Chen et al. [175]. Loss-of-function mutation of PPARγ was noted in some patients with colon adenocarcinoma [175,176]. Curcumin was found to activate PPARγ and suppressed the growth of both HT-29 colon cancer cells and Moser cells following inhibition of *EGFR* and cyclin D1 expression [175]. The activation of PPARγ signal transduction pathway inhibits the HT-29 colon cancer cell growth and suppress colorectal carcinogenesis, which was observed via an in-vivo study [177].

### 5.6. Activator Protein-1 (AP-1)

AP-1 is another transcription factor, with a structure of heterodimer that consists of protein belonging to c-Jun, c-Fos, and activating transcription factor (ATF), a subunit that attaches to a common DNA site, as the AP binding site [178]. Upon activation, AP-1 binds either to the TPA response element [16] or cAMP response element [179] in the promoter and enhancer region of the genes responsible for cell proliferation, survival, differentiation, cell migration, angiogenesis, metastasis and apoptosis, causing an increase in the expression [178,180]. In colorectal cancer, increased activity in AP-1 is linked to *KRAS* mutation, MSI-activation of Wnt/β-catenin pathway and truncated *APC* gene. Other than *AP-1* being the target gene of β-catenin, the association between AP-1 and CRC formation was indirect as it might involve activation of mitogen-activated protein kinase (MAPK) [161,181,182] and JNK pathways [181,183]. β-catenin, MAPK and JNK induce activation of c-myc, c-Jun and cyclin D1, thus promoting carcinogenesis. Curcumin, on the other hand was found to decrease AP-1 activity in colon cancer line HT-29 at higher concentration while increased AP-1 activity was noted at a lower concentration [184]. A similar finding was reported by Collet et al. [185], whereby curcumin at higher dose induces activation of AP-1, phosphorylation of c-Jun as well as activation of JNK in HCT-116. Curcumin, therefore may able to regulate apoptosis in CRC either by acting as AP-1 inhibitor or AP-1 enhancer depending on the dosage or the cell type [185,186].

### 5.7. Phosphatidylinositol 3-Kinase/Protein Kinase B (PI3K/AKT)

Phosphatidyl-inositol 3-kinase (PI3K) is an intracellular lipid which plays a vital role in cell regulation and cancer development [187]. The key components of the PI3K/AKT pathway are PI3K, AKT, glycogen synthase kinase 3 (GSK3-β), mammalian target of rapamycin (mTOR), S6 ribosomal protein (S6RP) and eukaryotic translation initiation factor 4E-binding protein 1 (4E-BP1) [188]. The regulatory (p85) and catalytic (p110) subunit of PI3K are found to be linked with cancer [189,190,191,192,193]. PI3K in an active state converts phosphatidylinositol (4,5)-bisphosphate (PIP_2_) into phosphatidylinositol (3,4,5)-trisphosphate (PIP_3_) followed by the binding of PIP_3_ to the pleckstrin homology (PH) domain of AKT/PKB, thus activating the AKT [194]. Activation of the AKT leads to inhibition of Bcl-2-associated death promoter (BAD) and Bax pro-apoptotic protein [187,190,195], suppression of p53-mediated apoptosis via Mdm2 phosphorylation, increased transcription of anti-apoptotic and pro-survival genes via NF-κB transcription factor [187,196], and induce activation of mTOR which is a group of protein associated with cancer metastasis [190]. Curcumin was found to regulate downstream apoptosis related genes (caspase 3, cytochrome C, Bax and Bcl-2) by suppressing PI3K/AKT pathway in human colon cancer cell lines, LoVo [197]. Although AKT protein levels were not affected by curcumin treatment, downregulation of p-AKT was observed. This is contradictory with CRC *PTEN*-deficient cells whereby curcumin increased the p-AKT expression [197]. Continuous activation of PI3K and AKT stimulate downstream signaling pathway, including cell proliferation and resistance towards apoptosis. The activation of PI3K/AKT, however, might be suppressed by tumor suppressor phosphatase and tensin homolog (*PTEN)*, which dephosphorylates PIP_3_ to PIP_2_ [195]. A decrease in PTEN expression was found in 46% of the adenomatous polyps, an early sign for CRC carcinogenesis [198,199]. Besides, Naquib et al. [200] reported the loss of PTEN occurred in 35% of CRC cases. Findings by Chen et al. [201] showed curcumin enhanced cytotoxicity against CRC *PTEN*-deficient cells. It was proposed that the loss of PTEN expression might lead to the alteration of cell cycle arrest pattern induced by curcumin. The alteration of the cell cycle pattern might be associated with the PTEN-regulated AKT/p21 signaling without the enhancement of apoptosis induction [201]. Increased in p21 expression was also observed upon curcumin exposure which leads to down-regulation of cyclin B1, Cdc2 and G2/M phase arrest in CRC *PTEN*^++^ cells [197,202]. However, opposite finding was observed in CRC *PTEN*-deficient cells whereby p21 expression was decreasing [197]. This is in correspond to the increase in p-AKT which able to phosphorylate p21, following subsequent degradation in the cytoplasm [203]. A decreased in p21 expression, reduced in cyclin D1 level and G0/G1 arrest was observed in CRC *PTENT*-deficient cells undergone curcumin treatment [197]. Moreover, about 60%–70% of human colon cancer involved with the activation of AKT signaling and dysregulation of PTEN [199]. Another study done by Johnson et al. [204] reported that p85α, AKT1, AKT2, p-mTOR ^Ser2448^, and p-p70s6K^Thr389^ are overexpressed in CRCs and are more prominent in left-sided CRC. Higher expression of p85α was also observed in stage IV of CRC [204]. Curcumin has also shown to exhibit potent radiosensitizing effect via inhibition of the PI3K/AKT/mTOR pathway in gut-specific endothelium cells [205]. In addition, Johnson et al. [206] observed the anti-proliferative effect of curcumin is via inhibition of mTOR signaling which includes the declining of mTOR, Raptor and Rictor levels accompanied with the induction of AKT (Ser 473) phosphorylation. The induction of AKT phosphorylation, however, may be attributed to a decreased in PHLPP1 phosphatase level, an inhibitor of AKT [206].

### 5.8. Cyclin D1

Cyclin D1 is a protein belongs to cyclin family which functions as regulators of cyclin-dependent kinases (CDKs). The role of Cyclin D1 includes the cell cycle transition from G1 to S phase [207], as a transcription factor [208,209,210], mitochondria biogenesis [211,212,213,214,215,216] and genomic instability [217], which may directly or indirectly involve in disruption of apoptosis [218] in CRC. Cyclin D1 overexpression and loss of function in p53 was observed in immuno-stained tissues samples of patients with primary colon carcinoma [219,220,221] may prevent CRC cells undergoing apoptosis. An elevated level of cyclin D1 in CRC [222] was found to be correlated with increased β-catenin due to the *APC* mutation [208,223]. Wangefjord et al. [224] reported cyclin D1 expression which is much lower in male compared to female is strongly associated with prolonged survival in male CRC. Another cohort study by Ogino et al. [219] also supported the correlation between elevated cyclin D1 level and prolonged survival of colon cancer patients. Curcumin has been found to down regulates cyclin D1 and induced G1 cell cycle arrest in HCT-116 colon cancer cells [225]. Cyclin D1 is commonly known to bind to either CDK4 and CDK6 forming active complex that further phosphorylates Rb at Ser^780^ regulating the transition from G1 to S phase [226]. Phosphorylation of Rb release E2 transcription factor (E2F) that regulates the expression of other genes needed for G1 to S phase transition. Upon activation of the E2F, cyclin E binds to CDK2 [227]. Moreover, curcumin has been reported to directly targets CDK2 leading to cell cycle arrest at G1 phase [225], thus inhibiting cells from further entering S phase. Inhibition of cell proliferation was observed in colon cancer cell lines HCT-116, HCT-115, and DLD-1 after curcumin exposure with reduction in CDK2 level [225]. Although curcumin was found to inhibit CDK1 activity, the inhibitory effect was more prone against CDK2 due to the stronger binding affinity. It is suggested that curcumin may interact with CDK2 through ATP pocket of CDK2. There is also association between enhanced apoptosis and increased in G2/M phase arrest. During G2 phase, increase in cyclin D1 level may affect the CDK1 activation. The activation of the CDK1 level however, is depends upon cyclin B1, growth factor and Ras activity which contribute to cell decision either arresting at G2/M or entering cell mitosis [228]. Cells arresting at G2/M phase may either undergo DNA repair mechanism or apoptosis. Findings by Su et al. [229] demonstrated that curcumin treatment on COLO-205 colon cancer cells up-regulates Wee1 and down-regulates Cdc25c, CDK1, and cyclin B1 and promoting entry into G2/M arrest. Wee1 serves as CDK1 inhibitor [230] while cyclin B1 serve as mitosis “on” switch whenever attached to CDK1 forming mitosis-promoting factor (MPF) [231], which explained curcumin as a G2/M arrest inducer, thus promoting apoptosis. In addition, curcumin also was found to regulate cyclin D1 via activation of PPAR-γ [175], inhibition of NF-κB [232] and AP-1 [184].

### 5.9. COX-2

Overexpression of COX-2 was noted in numerous tumors including colorectal cancers [233,234,235,236,237,238,239,240] and are linked to poor prognosis [241,242,243,244]. Resistance towards apoptosis in colon epithelial cell during colon carcinogenesis may attribute to the COX-2 overexpression [245]. The mutations in *BRAF* and *KRAS* oncogenes which have been found in approximately 10–20% and 35–42% of sporadic colorectal cancers respectively [246,247,248], has been shown to contribute to the up-regulation of COX-2 [249,250]. The mutated *KRAS* may activate MAPK/ERK kinase/ERK pathway which act together with PI3K/AKT/PKB pathway leading to post-transcriptional stabilization of COX-2 mRNA [251,252]. Increased COX-2 transcription or mRNA stability due to the oncogenic *RAS* and the loss of function of the *APC* tumor suppressor gene may occur via signal transduction pathway of Wnt/APC, RAS signaling, ERK, p38 MAPK, and AKT/PKB (protein kinase B) [250]. Moreover, COX-2 is down-regulated by wild type *APC* [253,254] but up-regulated by both nuclear β-catenin accumulation and Ras signal transduction pathways [250]. The β-catenin, a transcription factor of both phosphoprotein enriched in astrocytes (PEA) family member and Wnt-1 pathway are believed to be the mediator of the link between APC and COX-2 [250,255,256]. CRC involving the distal (left) colon is higher in COX-2 mRNA expression, more aggressive and are prone to have mutations in *APC*, *TP53,* and *KRAS* genes [254,256,257,258,259,260]. Studies revealed that elevated levels of COX-2 in CRC is more likely to occur in the aggressive and advanced stage [261,262,263,264]. About 77% of colorectal carcinoma has been identified with elevated expression of COX-2 in comparison to the adjacent normal mucosa [265]. Several studies have demonstrated that curcumin was able to repress COX-2 expression in CRC [266,267,268]. The downregulation of COX-2 by curcumin was mediated through NF-κB [130,267]. Plummer et al. [143] reported that curcumin able to modulate the signaling pathway, which regulates the stability of the NF-κB sequestering protein, IκB leading to inhibition of *COX-2* expression. Curcumin hindered the tumor promoter-mediated NF-κB transactivation by suppressing the NIκ/Iκκ signaling complex which might involve with Iκκ α/β in human colon cancer cells [143]. Curcumin was also found to exhibit apoptotic effects on HT-29 colon cancer cells by reducing COX-2 expression and apoptosis-related kinase pAKTand up-regulating p-AMP protein kinase (AMPK) expression [269]. It is suggested that AMPK, a metabolic sensor of cellular energy status which is activated during increase levels of AMP and depletion of cellular ATP may control apoptosis [270] through inhibition of AKT and COX-2 expression [269]. AMPK may serve as apoptotic molecules and its activation by phytochemicals such as CGCG, resveratrol, and capsaicin has been observed to be associated with apoptosis induction in cancer cells [271].

COX-2, also known as prostaglandin H synthases-2 or PTGS2 [272] converts arachidonic acid (AA) into prostanoids, which includes prostaglandins (PGs) and thromboxanes (TXs) [273]. Overexpression of COX-2 correlates with the decrease in intracellular AA and increase in the prostaglandin E2 (PGE_2_) production [274], may promote angiogenesis, tumor formation, metastasis, inhibition of apoptosis [275], suppression of the immune response, and induce precursor activation of carcinogenic substances [234]. PGE_2_ was found as the most abundant product of COX-2 during colorectal carcinogenesis [274] and lymph nodes metastasis [234]. Correlation between the PGE_2_ levels and tumor growth has been reported in Familial Adenomatous Polyposis (FAP) patients and HCA-7 colon cancer cells [276]. Increased in PGE_2_ level inhibits apoptosis through up-regulation of the Bcl-2 [242,272,277,278], modulation of the pro- and anti-apoptotic protein as well as downregulation of tumor suppressor gene via secretion of cytokines [272]. Overexpression of COX-2 in HCT-15 colon cancer cells may also suppress the DR5 transcription, suggesting that the COX-2 able to regulate the apoptotic extrinsic pathway [279]. Numerous evidence from different sources of studies in-vitro, in-vivo, or clinical data demonstrated that selective COX-2 inhibitors may reduce prostaglandin production and the risk of CRC [242]. Administration of curcumin in advanced CRC patients inhibits lipopolysaccharide (LPS)-induced PGE_2_ [280], decrease in malondialdehyde-DNA M1G adduct (MDA) and reduce COX-2 level in colorectal cancer tissues [281]. A reduction in PGE_2_ production was also observed in human blood samples [282]. However, in contrast to other studies, curcumin supplementation was unable to reduce COX-2 protein level [283]. In addition, curcumin intake in advance CRC may correspond to dose-dependent PGE_2_ inhibition [284]. PGE_2_ may acts through transmembrane protein receptor, EP4 leading to mitogen-activated protein kinases (MAPK) activation and thus, inhibits apoptosis [277,278]. It has been reported that the number of colorectal adenomas was inversely related to the apoptosis rates and COX-2 inhibitors treatment was found to correlate positively with the apoptosis reduction [270,285]. However, the COX-2 inhibitors such as NSAIDs do not inhibit the peroxidase function of the COX-2 enzyme, which is noted to exhibit both cyclooxygenase and peroxidase activities. Curcumin on the other hand, capable in inhibiting both the peroxidase and cyclooxygenase activities of the COX-2 enzyme [286]. Moreover, curcumin was found to directly inhibit the COX-2 expression at the mRNA and protein level in HT-29 human colon cancer cells. Another study by Shehzad et al. [287] demonstrated curcumin induced apoptosis are correlated with suppression of COX-2, PGE_2_, MMP-2 and MMP-9, Ca2þ mobilization, and AP-1 activation. In addition, supplementation of curcumin in the mouse model with severe colon inflammation also reduces TNF-α, IFNγ, COX-2, and inducible nitric oxide synthase (iNOS) levels in colon tissue [288,289].

### 5.10. TP53

The guardian of the genome, *TP53* tumor suppressor gene was found mutated in 85% of colorectal cancers [290] with 70% of the *TP53* mutations were linked to adenoma-to-carcinoma progression, an aggressive subset of CRC [291,292]. About 75% of CRC patients display common deletion of genetic material on chromosome 17, loci for *TP53* gene (17p13.1) [292]. *TP53* may induce apoptosis extrinsic or intrinsic pathway triggered via cellular stresses from nutrient deprivation and hypoxia, DNA damage, proliferation and cell survival [293,294]. Curcumin, a yellow pigment of diarylheptanoid isolated from rhizome of *Curcuma longa* has been extensively studied on its apoptosis inducing capabilities on various cancer cell lines involving p53 modulation [295,296,297]. p53 upon activation, triggered the mitochondria apoptotic pathway by acting as transcription factor that regulates the expression of pro-apoptotic Bcl-2 family member mainly Bax, BH3,phorbol-12-myristate-13-acetate-induced protein 1 (NOXA), and PUMA, and down-regulates the anti-apoptotic Bcl-2, Bcl-xL, and inhibitors of apoptosis proteins (IAPs), which includes Survivin [298]. The suppression of IAP-Survivin may directly increase caspase activity and promote apoptosis [298]. Curcumin has been found to promote cytochrome C release accompanied by increased expression of p53 and Bax as well as reduction of Bcl-2 and Survivin [299]. It has been reported that p53 may also increase expression of apoptosis effector components such as PTEN, Apaf-1, PERP [300,301], caspase 6 [302,303], and ferredoxin reductase (FDXR) that promotes the increase in reactive oxygen species (ROS) [304]. ROS promote apoptosis by either regulating mitochondria membrane permeability or through MAPK via c-Jun N-terminal kinase (JNK) and p38 signaling pathway. Once activated, the JNK and p38 translocate to the nucleus and initiate apoptosis [305]. Reduction in ROS production by disruption of *FDXR* gene or addition of exogenous antioxidants reduce p53 mediated apoptosis in colon carcinoma cells treated with 5-FU [306]. Curcumin however displays the ability to induce apoptosis in HCT-116 colon cancer cells carrying defects in p53 expression mediated by elevated level of superoxide anion. Increased level of superoxide anion due to curcumin exposure promotes oxidative stress despite the absence of functional p53 [307].

Moreover, curcumin has been shown to induce apoptosis in both ways, either p53-dependent or p53-independent in various cell lines including colon carcinoma [308,309]. Curcumin also displays the ability to signal switch the MEK/ERK proliferative signaling to p38 MAPK/JNK1 pro-apoptotic pathway leading to phosphorylation of p53, transactivate BAX and Bcl-2 binding component 3 (*PUMA*) genes, promoting cell death in human CRC [310]. In addition, curcumin was found to increase in p53 expression in colon cancer cells [115,311,312], as well as down-regulates the survival genes *EGR-1* (Early growth response), c-myc, Bcl-2, and Bcl-xL [313], thus promoting apoptosis. Elevated level of p53 expression, higher number of apoptotic cells, increased body weight and decreased in TNF-α serum level was observed in a pilot study involving CRC patients [312]. Similar findings were also observed in other clinical trial involving CRC patients after diagnosis, and before undergone surgery [282,312,314]. Increase in p53 expression by curcumin may affect the survival pathway. p53 may switch off the survival pathway that overrides apoptosis such as PI3 kinase/AKT pathway. p53 suppresses the PI3K/AKT survival pathway via increase expression of the PI3K inhibitor PTEN [100]. p53 might also trigger apoptosis whenever there are excessive proliferation signals of oncogenes such as *MYC*, the adenovirus early region 1A (*EA1*) and *E2F* as protection against neoplastic transformation of abnormal cells [294].

Watson et al. [307] demonstrated up-regulation and activation of p53 together with increased expression of p53-responsive genes *p21*, *PUMA*, and *BAX* upon curcumin exposure on HCT-116 carrying wild type *TP53* and HT-29 with *TP53* mutation. In contrast, curcumin treatment on HCT-116 with loss of p53 function did not show increased in PUMA and BAX expression [307], but with limited accumulation of p21 [117], an indication of cell death mediated by other pathway [117,315]. Loss of p53 function has shown in in-vitro study to reduce chemosensitivity in colorectal cancer cells towards the 5-FU [316,317]. However, the opposite finding was observed in Stage III [318] and Stage IV [319] of colorectal cancer patients carrying p53 overexpression whereby it was shown to be resistance to 5-FU chemotherapy. Multiple factors determine the outcome stimulated by p53 activation either promoting or impede apoptosis. Cell and tumor types, cellular microenvironment and intracellular signals may have some influences in the p53 activation or p53 aberration outcome [317,320]. Study done by Dasiram et al. [321] however, demonstrated curcumin able to induce apoptosis in COLO-320DM colon cancer cells (Dukes’ type C stage) carrying mutated *TP53* and arresting the COLO-320DM human colon adenocarcinoma at G1 and S phases. Besides, treatment of curcumin on colorectal carcinoma, induces caspase 3 mediated apoptosis by decreasing expression of mutant p53 and decreasing pre-mRNA processing factor 4b (Prp4B) in a dose and time-dependent manner [322]. While p53 activation may up-regulates the expression of some death receptors (DR) such as Fas (CD95/APO-1), DR5 (TRAIL-R2), and PIDD (p53-induced protein with domain) as well as the BH3 only protein (BID) that couples the extrinsic pathway to activate the intrinsic pathway [323], the role of curcumin in inducing apoptosis mediated by increase expression of death receptor via p53 has not yet been reported.

### 5.11. ROS

Reactive oxygen species (ROS) are unstable and highly reactive small molecules which are normally the by-products of normal cellular oxidative process such as enzymatic reaction, electron transport chain or mitochondria phosphorylation [324], that includes singlet oxygen (O^−^), hydroxyl radical (OH^−^), hydrogen peroxide (H_2_O_2_) and super anion radical (O_2_^−^) [325,326]. Apart from mitochondria as the main source of ROS (90%) [327], the nicotinamide adenine dinucleotide phosphate (NADPH) oxidase (NOS) is another source of intracellular ROS that usually response to stress [328]. The role of ROS in cellular physiology process is depends on its concentration. In normal condition, the production and scavenging of ROS are kept in balance thus enabling the ROS to function as vital secondary messages. This regulates multiple physiological regulation processes that include gene regulation, cytokines, growth synthesis, cell proliferation, migration and differentiation which are important in maintaining cellular homeostasis [329]. The imbalance of ROS production with its scavengers may lead to oxidative stress and oxidative damage [330]. The accumulation of ROS at moderate level leads to cell damage, DNA mutation and inflammation, thus promoting cancer formation [330] while excessive ROS promote apoptosis [331]. The damage of nucleic acid, proteins and lipids due to oxidation or lipid peroxidation by ROS may alter the integrity of mitochondrial membrane potential, leading to the activation of pro-apoptotic protein Bax and subsequently, the release of cytochrome C. The release of cytochrome C will activate the mitochondria-mediated apoptotic pathway [331,332]. However, uncontrolled and continuous ROS overproduction may cause gut barrier dysfunction and release of inflammatory cytokines [330] that may lead to CRC development [333,334]. It has been demonstrated that patients with IBD (inflammatory bowel diseases), which was known to have elevated ROS level [335,336,337], may have 2–3-fold higher risk in developing CRC [338,339,340]. In addition, ROS-induced DNA damage and genetic mutations such as single and double strand breaks, and other mutations occurring in *KRAS*, *BRAF, APC*, and *p53* are associated with CRC [341,342,343,344]. Malondialdehyde (MDA) and 4-hydroxy-2-nonenal (HNE), which are products of lipid peroxidation were also found in high concentrations in CRC tissues [345]. This may indirectly induce loss of APC function and reduced β-catenin degradation [234,346,347,348], inhibits apoptosis and promotes CRC carcinogenesis. In addition, higher level expression of NADPH oxidase 1 (NOX1) also has been identified in the colon [349,350]. The NOX1-derived ROS generation stimulates Wnt/β-catenin and Notch signaling pathway thereby, enhancing cell proliferation [351,352] and gain resistance towards apoptosis. Redox modifications of protein cysteine residues in CRC development may involve with signaling pathway and transcriptional factors modulators [353] such as Sp [342], NF-κB [354,355,356], p53 [357,358,359,360], HIF-1α [361,362], and Nrf2 transcription factor [342,363,364,365], c-Myc [351], MAPK cascade [366,367,368], PI3K/AKT [369,370,371], and janus kinases/signal transducer and activator of transcription proteins (JAK/STAT) signaling pathway [358,372].

Curcumin, a promising anti-cancer agent has been found to induce apoptosis by increased ROS generation hence inducing oxidative responds and disruption of membrane mitochondria permeability in cancer cells including CRC [373,374]. Curcumin induces ROS that activates apoptotic mechanism that might contribute to the improvement of redox status via reduction of iNOS expression and inhibition of arginase activity [375]. Moreover, curcumin able to change the redox status that affects mitochondrial permeability transition through suppression of mitochondrial NADP^+^ dependent isocitrate dehydrogenase (IDPm) by targeting Cys379 residues and repressing NADPH generation [376]. It also has been reported that induction of ROS might be linked with thiol redox signaling cascade involving phosphorylation of PKCδ and PI3K pathway, up-regulation of growth arrest and DNA damage 153 (GADD153) and reduction of intracellular glutathione [377]. Other studies have also observed induction of ROS activates ROS-independent mitochondrial apoptotic pathway in mutated p53 and Smad2, a mediator of TGF-β in colon adenocarcinoma HT-29 [374]. The activation of ROS might also induce specific protein (Sp) repressor ZBTB10 and ZBTB4 leading to downregulation of Sp1, Sp3, Sp4 transcription factor and Sp-regulated genes [153]. Induction of apoptosis by curcumin seems to be cell selective in-vitro and may depends on the ability of the cells to generate superoxide radical and the Hsp70 (heat shock protein-70) expression level [378,379]. An increase in Hsp70 prevents induction of apoptosis by curcumin as Hsp70 functions as a chaperon and protects cells through an antioxidant mechanism by stabilizing endogenous antioxidant-glutathione [378,380]. Curcumin at higher concentration induced apoptosis in COLO-258, but not in SW620 even though increased expression in superoxide anions observed in both cell lines. It was suggested that inhibition of apoptosis in SW620 was due to the insufficient concentration of superoxide anions and higher concentration of Hsp70 level. Curcumin may induce apoptosis independent of cytochrome C [378,381] involving different signaling pathways in the apoptotic cascade and the level of Hsp70 was not sufficient enough to show protection against apoptosis subjected to ROS in COLO-258 cells [378]. However, Watson et al. [307] reported that curcumin increases superoxide anion production leading to p53-independent apoptosis in HCT-116 colon cancer cells. Apoptosis induction via the curcumin-ROS-associated mechanism may also involve JNK activation and ceramide associated pathway [67]. There is also possible involvements of rapid ROS generation resulting in an increased level of Ca^2+^ [117], activation of caspase 3, downregulation of mutated p53, and Prp4B signaling pathway [322]. Prp4B is a spliceosomal factor in pre-mRNA splicing and RNA maturation. Mutation of Prp4 disrupts cell cycle transition and accumulation of pre-mRNA [322].

### 5.12. Curcumin Induce Apoptosis via Mitochondria Independent Pathway-Endoplasmic Reticulum (ER) Stress

There is evidence that apoptosis might be induced by mitochondria independent pathway which may include the endoplasmic reticulum (ER) stress-induced apoptosis [382,383]. Other than site for calcium (Ca^2+^) storage, ER functions as checkpoint control by ensuring the quality, correct folding and secretion of the newly synthesized protein. It served as vital “detector” of cellular stress and may halt protein synthesis in order to gain cellular homeostasis [384]. Imbalance of the ER homeostasis may induce ER stress. Disruptions of the ER homeostasis might include the accumulation of the unfolded proteins in the ER [385], hypoxic conditions, low pH, low glucose, and low nutrient supply [386], which triggers the UPR (unfolded protein response). The prolonged ER stress and continuous activation of UPR might trigger apoptosis [387,388,389] mediated by the UPR signal across the ER membrane together with the release of calcium into the cytoplasm [384] and eventually affect calcium concentration in mitochondria via mitochondria-associated membranes (MAMs) [390]. However, instead of promoting apoptosis [391], cancer cells used to adapt to the new environment and able to escape apoptosis [385] via ER chaperon Bip, also known as glucose-regulated protein 78 (GRP78) [392,393,394,395,396]. It was found that increased expression of GRP78 was observed in colon cancer [397]. Elevated level of GRP78 has been associated with CRC of poor survival rate, tumor invasion, higher pathologic grade and recurrence risk [398]. Overexpression of GRP78 was located on various colon cancer cell surfaces such as HT-29, SW480, SW620, DLD1, and LoVo, may promote CRC cell migration and invasion [399]. GRP78 has also been suggested as a novel predictive biomarker for CRC [400].

Curcumin which is found abundant in turmeric exerts its antitumor activity via apoptosis induction [401]. Curcumin treatment on HT-29 colon cancer cells increased the caspase 12 expression and calpain level. It has been reported that apoptosis may also be activated via the ER-specific apoptotic pathway involving caspase 12 activation [402,403,404]. Calpain, a Ca^2+^ dependent cysteine protease, suppressed calpastatin an endogenous inhibitor of calpain and induce apoptosis [405]. The induction of apoptosis is due to the calpain/caspase 12 apoptotic pathway and also disturbance in the calcium homeostasis [405,406]. Curcumin induces the release of cytochrome C through the increase of mitochondria Ca^2+^. It was suggested that the mechanism of apoptosis regulation might involve the transfer of Ca^2+^ from ER to mitochondria, suppression of the sarco/endoplasmic reticulum Ca^2+^ATPase (SERCA) pump and decrease in mitochondrial membrane permeabilization (MMP) [71]. Nakamura et al. demonstrated that ER and mitochondria are closely associated, whereby Ca^2+^ released from ER assembles in mitochondria [407]. Increased level of intracellular free Ca^2+^ may induce activation of calpain. Upon activation, calpain migrates from the cytosol to the membrane and cleave pro-caspase 12 generating active caspase 12 [408] which later may directly activate caspase 3 [405]. Activation of caspase 3 may also involve with the activation of caspase 9 [409]. The caspase cascade event involving caspase 12 activation may trigger post adaptive UPR activation which involves pro-apoptotic C/EBP homologous protein (CHOP) transcription factor and inositol-requiring enzyme (IRE1) [410]. Overexpression of CHOP, which is also known as growth arrest and DNA damage-inducible gene 153 (*GADD153*), has been reported to induce cell cycle arrest and apoptosis by regulating the pro- and anti-apoptotic genes including *DOCs* (for downstream of CHOP), *BCL2*, *TRB3* (tribbles-related protein 3), and *GADD34* [411]. Increased level of GRP78, which was observed in colon cancer cell [397,398,399] functions as chaperone may dissociate from its conformational binding state of the transmembrane receptor protein kinase RNA like endoplasmic reticulum kinase (PERK), IRE1 and activating transcription factor 6 (ATF6). These event together with the decreased in IRE1 prevent apoptosis in CRC. IRE1β was found to be decreased in CRC tissues and might be associated with the clinical features of CRC patients [412]. In contrast, activated IRE1α might promote apoptosis via ER stress-induced c-Jun amino-terminal kinase (JNK activation [413] and caspase 12 activation by binding to tumor necrosis factor (TNF) receptor-associated factor 2 (TRAF2) [402,404]. Curcumin is a hydrophobic polyphenolic compound able to penetrate into the cytosol through the plasma membrane [405] was found to induce ER-stress-mediated apoptosis in HT-29 colon cancer cells via increased expression of CHOP, JNK, cytochrome C release and FADD [71]. Moreover, curcumin was shown to enhance apoptosis activity and increase efficiency of DNA repair during in-vivo study. The enhancement of apoptosis activity was mediated by GADD153 and X-ray repair cross-complementing protein 1 (XRCC1 [414]. Curcumin has also been reported to up-regulate GADD153 via glutathione modulation [377]. However, the mechanism of curcumin’s role in ER-apoptosis induction via CHOP in colon cancer remains unclear and needs further investigation.

## 6. Future Target of Curcumin in Apoptosis

Curcumin has been demonstrated to have the ability in inducing apoptosis despite the limitation in data showing its anti-cancer properties in CRC. While curcumin was proven to be safe in clinical trials, the efficiency of curcumin in inducing apoptosis is limited due to its low bioavailability and poor absorption by the GI tract. Further preclinical and clinical trials on curcumin with improved bioavailability and absorption either by modification of its side chain or with different mechanisms of delivery systems such as liposomal, nanoparticle, and adjuvants are needed to further explore the maximum potential of curcumin as CRC anti-cancer agent in promoting apoptosis.
